# Editorial: Anakoinosis: An Innovative Anticancer Therapy Targeting the Aberrant Cancer Tissue Homeostasis

**DOI:** 10.3389/fphar.2021.779021

**Published:** 2021-10-07

**Authors:** Daniel Heudobler, Albrecht Reichle, Lina Ghibelli

**Affiliations:** ^1^ Department of Internal Medicine III, Hematology and Oncology, University Hospital Regensburg, Regensburg, Germany; ^2^ Department Biology, Universita’ di Roma Tor Vergata, Rome, Italy

**Keywords:** anakoinosis, shear stress, DNA gels, gold nanorods, Hodgkin’s lymphoma, acute lymphoblastic leukemia, non-small cell lung cancer, graft versus host disease

## Targeting the Aberrant Cancer Tissue Homeostasis

The aim of anakoinosis is “tissue editing,” meaning that bioactive, regulatory acting principles are combined to re-establish tissue homeostasis at primary and metastatic tumor sites by communicatively reprogramming tumor tissue and cell recruitment (Heudobler et al., 2019). Pro-anakoinotic therapies combine regulatorily active drugs, even those with poor or no monoactivity, and may positively supplement classic targeted therapies, as exemplified in relapsed or refractory (r/r) classic Hodgkin’s lymphoma (cHL) with the successful introduction of a target of rapamycin (mTor) inhibitor (Heudobler et al., 2018a; Lüke et al.).

This Research Topic highlights differential clinical outcome characteristics in three histologically completely different neoplasia as a response to “tissue editing” when treated either with pro-anakoinotic approaches (in case of r/r cHL and r/r non-small cell lung cancer, NSCLC) or accidentally initiated by severe fungal infection and reduced intensity induction chemotherapy in acute lymphoblastic leukemia (ALL) (Lüke et al.; Lüke et al.; Heudobler et al.). The clinical responses outline the possibility to specifically activate unique tumor tissue dynamics in response to pro-anakoinotic effectors, either by combining differently acting biomodulatory drugs, or associating DNA damage with fungal infection.

DNA damage response following “one shot” chemotherapy in ALL seems to serve as a homeostatic trigger in the case of parallel fungal infection, presumably supervising and amplifying immunological response by the innate immune system, thereby preventing both early relapse and persistence of minimal residual disease, as indicated by long-term continuous complete remission in both described ALL cases (Lüke et al.).

In r/r NSCLC, explorative studies of the randomized trial provide strong hints that pre-treatment with combined biomodulatory therapy may be the basis for successful consecutive immune checkpoint inhibitor (ICPi) therapy: even though the progression-free survival rate of the biomodulatory treatment arm is significantly inferior to that of the ICPi arm, it, however, exerts tissue modifications that render successive ICPi more efficacious. These results stimulate the hypothesis that the addition of ICPi to the biomodulation could be beneficial (Heudobler et al.).

The immune-modulatory acting MEPED schedule for r/r cHL resulted in pivotal outcomes in six cases. Whether a patient is frail and does not respond to reduced standard first-line therapy, or patients are r/r after autologous hematopoietic-stem-cell transplant (autoHSCT), or even allogeneic hematopoietic-stem-cell transplant (alloHSCT), MEPED may induce complete PET negative remission. The remissions could be maintained by alloHSCT. But even after discontinuation of MEPED in a frail patient, CR continued without any HSCT. Like in NSCLC, a possible beneficial supplementation of the immune-modulatory schedule could be ICPi therapy (Lüke et al.).

## Novel Technical Approaches to Study the Biological Basis of Anakoinosis for Sustainable Drug Development

The use and development of pro-anakoinotic therapies are not restricted to neoplasia. Reprogramming of dysregulated homeostatic pathways, stress response, autophagy, redox and metabolic pathways, and of homeostatic immune regulatory circuitries is generally of pivotal interest, irrespectively of the origin of disease (Kumari et al.; Heudobler et al., 2019).

A comprehensive overview of metabolic changes mediating homeostatically dysbalanced immunologic response during graft versus host disease (GVHD) has been given by the group of Kumari et al. The large series of dysregulated metabolites in GVHD suggest that pro-anakoinotically active drug combinations could contribute to improve or inhibit GVHD in addition to established targeted therapies. In mouse GVHD, for example, the nuclear receptor agonist rosiglitazone may prevent GVHD, an approach that should be studied further in context with additional biomodulators (Song et al., 2012; Heudobler et al., 2018b).

Besides therapeutic modulation of cell metabolism, immune response, or tumor-associated inflammation in neoplasia, as exemplified in cHL, NSCLC, and ALL or GVHD, reprogramming the mechanical properties of tumor and adjacent stromal cells may be an important new therapeutical direction (Favero et al.; Lüke et al.; Lüke et al.; Heudobler et al.). *In vitro* studies by Del Favero identify novel biological paths induced by bio-physical circumstances, such as shear stress, and give a first hint on how to target respective biologic structures for reprogramming tumor functions. Drugs used in pro-anakoinotic schedules, like mTOR inhibitors, e.g., in cHL, may reprogram a response to shear stress, as experimentally shown (Favero et al.; Lüke et al.).

For successful development of combination therapies in the field of anakoinosis, the detailed description of complex tissue functions supporting disease-related, pathophysiologically dysbalanced homeostasis is necessary. This message clearly emerges from the contributions on GVHD, and from the mechanistic analysis of how T24 bladder cancer cells cope with fluid shear stress by modifying their shape (Kumari et al.; Favero et al.). The ALL studies highlight that clinical courses of malignant diseases may cause tissue alterations that render therapeutically accessible homeostatic disbalances previously hidden (Lüke et al.).

Successful pro-anakoinotic biomodulatory therapies in resistant neoplasia of quite different histological origins explicitly emphasize that diagnostics based on methods beyond tumor genomics are of pivotal interest for selecting therapeutical directions to achieve tumor control, particularly in metastatic, refractory disease. This would give a logical framework that allows for selecting drug combinations among the huge amount of available bioactive drugs, and for further pharmacological drug development (Muqaku et al., 2017; Lattuada et al.; Heudobler et al., 2018a).

Development and evaluation of biomodulatory drugs is already ongoing (Lattuada et al.; Baer-Dubowska et al., 2021). Dihydrotanshinone, a naturally occurring compound has been presented in the Research Topic as an inhibitor of hepatocellular carcinoma (HCC) growth by suppressing the JAK2/STAT3 pathway. Systemic therapy of non-resectable HCC still represents a field of medical need (Hu et al.).

A novel technology for the evaluation of systems biological drug interactions, relevant for anakoinosis research, has been presented by Lattuada et al. In the future, DNA-gels could be helpful for studying resetting cellular dysregulated homeostasis in disease, particularly in cancer, including immune activation, inflammation control, or *trans*-differentiation processes of malignant cells within their cellular environments. As a preclinical platform for evaluating novel drug combinations with pro-anakoinotically active drugs, 3D tissue-like DNA-gels could be implemented, incorporating cell spheroids for the assembly of a novel kind of cellular matrix (Lattuada et al.).

Gold nanorods, currently studied as potential therapeutics in photothermal therapies, may also contribute to evaluating pathophysiology in tumor tissues, and as such the impact on tissue homeostasis, by identifying specific binding sites of gold nanorods in single-cell compartments (Liao et al.).

In summary, this research topic provides a novel clinical collection of pro-anakoinotic approaches modifying dysregulated homeostasis in quite different ways for long-term tumor control or even healing of refractory tumor disease. Hopefully, pre-clinical *in vitro* test systems, as indicated by the Research Topic, may promote the exploration of biomodulatory drugs derived from different pharmacological tools for designing pro-anakoinotically acting combination therapies, particularly for overcoming therapeutic problems with refractory neoplasia and complex non-malignant diseases associated with tissue dysregulated homeostasis (Kumari et al.; Lüke et al.; Lüke et al.; Heudobler et al.; Vogt et al., 2006).

## References

[B1] Baer-Dubowska W. Narożna M. Krajka-Kuźniak V. Violetta (2021): Anti-Cancer Potential of Synthetic Oleanolic Acid Derivatives and Their Conjugates with NSAIDs. In: Molecules 26 (16), S. 4957. 10.3390/molecules26164957 34443544 PMC8398353

[B2] Heudobler D. Rechenmacher M. Lüke F. Vogelhuber M. Pukrop T. Herr W. (2018b). Peroxisome Proliferator-Activated Receptors (PPAR)γ Agonists as Master Modulators of Tumor Tissue. Int. J. Mol. Sci. 19 (11). 10.3390/ijms19113540 PMC6274845 30424016

[B3] Heudobler D. Lüke F. Vogelhuber M. Klobuch S. Pukrop T. Herr W. (2019): Anakoinosis: Correcting Aberrant Homeostasis of Cancer Tissue-Going beyond Apoptosis Induction, Front. Oncol. 9, S. 1408. 10.3389/fonc.2019.01408 31921665 PMC6934003

[B4] Heudobler D. Rechenmacher M. Lüke F. Vogelhuber M. Klobuch S. Thomas S. (2018a): Clinical Efficacy of a Novel Therapeutic Principle, Anakoinosis, Front. Pharmacol. 9, S. 1357. 10.3389/fphar.2018.01357 30546308 PMC6279883

[B5] Muqaku B. Eisinger M. Meier S. M. Tahir A. Pukrop T. Haferkamp S. (2017): Multi-omics Analysis of Serum Samples Demonstrates Reprogramming of Organ Functions via Systemic Calcium Mobilization and Platelet Activation in Metastatic Melanoma. Mol. Cell Proteomics 16 (1), S. 86–99. 10.1074/mcp.M116.063313 27879288 PMC5217785

[B6] Song E. K. Yim J. M. Yim J. Y. Song M. Y. Rho H. W. Yim S. K. (2012): Rosiglitazone Prevents Graft-Versus-Host Disease (GVHD). Transpl. Immunol. 27 (2-3), S. 128–137. 10.1016/j.trim.2012.09.001 22982856

[B7] Vogt T. Coras B. Hafner C. Landthaler M. Reichle A. (2006): Antiangiogenic Therapy in Metastatic Prostate Carcinoma Complicated by Cutaneous Lupus Erythematodes. Lancet Oncol. 7 (8), S. 695–697. 10.1016/S1470-2045(06)70798-7 16887488

